# Rotavirus mucosal immunology: insights and research priorities from an international convening in Liverpool, March 2024

**DOI:** 10.1038/s41541-026-01505-w

**Published:** 2026-06-19

**Authors:** Christina Bronowski, Chikondi A. Mwendera, Carl D. Kirkwood, A. Duncan Steele, Michelle J. Groome, Khuzwayo C. Jere

**Affiliations:** 1 Institute of Infection, Veterinary and Ecological Sciences, Department of Clinical Infection, Microbiology and Immunology, University of Liverpool, Liverpool, UK; 2 Enteric, Diagnostics, Genomics and Epidemiology (EDGE), Gates Foundation, Seattle, WA USA; 3 Medical Research Council Vaccines and Infectious Diseases Analytics Research Unit, Faculty of Health Sciences, University of the Witwatersrand, Johannesburg, South Africa; 4 Department of Medical Laboratory Sciences, School of Life Sciences and Allied Health Professions, Kamuzu University of Health Sciences, Blantyre, Malawi

**Keywords:** Diseases, Immunology, Microbiology

## Abstract

Oral Rotavirus vaccines have reduced disease globally but multiple factors in low- and middle-income countries (LMICs) diminish vaccine effectiveness and disease burden remains high. A convening of experts reviewed advances in mucosal immunity, disease model systems, lessons from other pathogens and the lack of correlates of protection, to identify future research priorities. This report summarises the meeting proceedings and outlines a roadmap for advancing rotavirus immunology and next-generation vaccine development.

## Introduction

Rotavirus remains a major cause of acute diarrhoea among children under five years of age^1–4^. Four oral rotavirus vaccines (ORVs), RotaTeq (Merck & Co, Inc., Whiteriver, PA, USA), Rotavac (Bharat Biotech International Ltd., Hyderabad, India), Rotarix (GSK, Rixensart, Belgium) and Rotasiil (Serum Institute of India, Pune, India), are currently prequalified by the World Health Organization (WHO). These vaccines are safe, and offer protection against severe disease after a two- or three-dose schedule^5–7^ ORVs have significantly reduced rotavirus disease morbidity and mortality, yet their efficacy and effectiveness is markedly lower in low-and middle-income countries (LMICs) compared to high-income countries, which has been comprehensively observed in clinical trials^8–12^, observed post rotavirus vaccine roll-out^13,14^ and modelled from real-world usage^15,16^. The reasons for this reduced effectiveness in LMICs are poorly understood, multi-factorial and likely include chronic malnutrition, concurrent infections, gut dysbiosis, environmental enteric dysfunction (EDD), altered gut microbiota, and maternal antibody interference^17–19^. Viral VP4-histo-blood group antigen (HBGA) interactions confer genotype-dependent resistance, with Lewis-negative and non-secretor phenotypes offering protection against common strains^20^. Maternal secretor status affects breast milk antibody levels, with secretor-positive mothers often reducing vaccine take^21^. These are some of the key determinants of ORV underperformance in LMICs^18,22–24^. Serum IgA remains a commonly used marker of oral vaccine performance; however, it does not represent a mechanistic correlate of protection (CoP)^17^. Measurement of total anti-RV IgA titres does not identify specific rotavirus protein target or the mechanisms underlying heterotypic protection^25,26^. Consequently, serum IgA may have limited applicability for evaluating non-replicating or parenteral vaccine platforms. Some studies have also assessed type-specific neutralizing antibodies, although these too provided an incomplete picture of protective immunity. Emerging insights from mucosal immunology highlights important roles of B and T cell responses, non-neutralizing antibodies (nNAbs), and tissue-resident memory (TRM) cells. For example, VP6-specific nNAbs can mediate intracellular neutralisation via TRIM21^27^, while T cell responses, although often transient and functionally limited, may contribute to longer-term protection through TRM populations^28^.

Strategies to improve vaccine performance include neonatal administration^29^, revised infant dosing schedules to minimize maternal antibody interference^24,30^, separating ORVs administration from oral polio vaccines (OPV), and implementing nutritional interventions to support gut health^31^. However, sustained investment in mucosal immunology and the development of innovative vaccine approaches—including parenteral, oral-parenteral combination strategies-, and next-generation technologies—will be essential to overcome current limitations and achieve durable global rotavirus control.

To address these challenges, this convening brought together experts in rotavirus and mucosal immunology to identify key gaps in our understanding of rotavirus-specific mucosal responses and to prioritise future research directions. Drawing on advances from other pathogens and emerging vaccine technologies, the meeting aimed to inform a strategic research agenda for funders to accelerate progress in the rotavirus vaccine field.

This report summarises key discussions, highlights cross-cutting gaps, and proposes a roadmap to guide future research on rotavirus immunity and vaccine development.

### Meeting overview

The Rotavirus Mucosal Immunology Convening took place in Liverpool, UK, from 25–26 March 2024, bringing together global experts in mucosal immunology, rotavirus biology, vaccinology, and clinical research. Sponsored by the Gates Foundation and co-hosted by the University of Liverpool and the University of the Witwatersrand, the meeting aimed to advance understanding of rotavirus immune responses and identify priority research areas to improve vaccine performance and guide the next-generation vaccine development, particularly for LMICs.

Opening remarks from Khuzwayo Jere (University of Liverpool) and Duncan Steele (Gates Foundation) emphasised the urgent need to optimise existing rotavirus vaccines and deepen mechanistic understanding of mucosal immunity to inform new vaccine strategies. The organizers highlighted the goal of defining three to five priority research areas for future investment, with a focus on the immune mechanisms that influence oral vaccine take and protection in vulnerable populations.

Over the two day-meeting, participants explored a wide range of topics spanning rotavirus mucosal immunology, lessons from other pathogens, emerging experimental models, and CoP.

The convening began with a review of current knowledge on rotavirus immunology, covering innate, humoral, and cell-mediated responses; functions of non-neutralizing antibodies; and the role of maternal antibodies and host genetics. Key evidence gaps were identified, including the lack of well-defined CoP and limited understanding of how host genetics, maternal secretor status, microbiome composition, and baseline inflammatory conditions affect ORV performance.

The scope then broadened to consider mucosal immunity to other enteric and respiratory pathogens, illustrating the value of cross-pathogen insights for refining of rotavirus vaccine strategies. Advances in understanding systemic infections, tissue-resident immunity, antibody effector functions, and mucosal secretions provided further perspectives on immune system complexity.

Participants also examined innovative model systems, including human intestinal organoids and controlled human infection models (CHIM) from Zambia. These platforms offer powerful tools for interrogating mechanisms underlying vaccine performance.

A dedicated session on CoP highlighted the multifactorial nature of immune signatures in enteric and respiratory diseases and underscored the need to integrate serological, mucosal, and cellular markers. Discussions also considered the implications for vaccine design, clinical endpoints, and trial platforms.

Throughout the convening, the panel discussions and structured round-table activities helped synthesize ideas and build consensus around emerging priorities. The next section summarizes key insights from each thematic area.

### Scientific themes

#### Current understanding of rotavirus mucosal immunity

The session on rotavirus mucosal immunity explored the complex interplay between epithelial and innate immune responses that shapes susceptibility to infection. Ben Lee (University of Vermont College of Medicine, USA) provided a comprehensive overview of the current state of knowledge in rotavirus immunology, highlighting that natural immunity develops progressively with repeated exposures. Rotavirus is a non-enveloped double-stranded RNA virus in the *Reoviridae* family that infects mature enterocytes at the tips of intestinal villi. The VP6 protein forms the middle capsid layer and defines the rotavirus group, while the outer capsid contains two structural proteins: VP7, a glycoprotein that determines the G-type, and VP4, a protease-sensitive spike protein that defines the P-type. In the presence of intestinal trypsin, VP4 is cleaved into VP8* and VP5* subunits, which mediate viral attachment and entry^32^.

The first infection with rotavirus is typically the most severe, whereas subsequent infections confer increasing protection against disease rather than sterilising immunity^33,34^. Although rotavirus-specific serum IgA remains the most widely used CoP, vaccine-induced responses in children living in LMICs are often reduced, partly due to interference from maternal antibodies^18,24^. This highlights the need for improved CoP, particularly in high-burden settings. However, progress is limited by the incomplete understanding of the precise immune mechanisms that confer protection against rotavirus^32,35^. Secretory IgA is the dominant mucosal antibody mediating intestinal protection, although in infants, it is also passively acquired from breast milk. IgM and IgG are present in the intestinal lumen, while peripheral blood contains mainly IgG (predominantly maternally transferred in utero), monomeric IgA, IgM, and small amounts of secretory IgA^32^. Both IgA and IgG titres increase with age and cumulative exposure through natural infection^33,36^. Serum rotavirus IgA is relatively easy to measure, correlates with duodenal IgA up to four months post-infection, and is unaffected by maternal antibodies or breast milk in some settings following acute gastroenteritis^37^. However, as a systemic marker, it likely serves as a non-mechanistic correlate of mucosal protection. While total serum IgA remains the best available CoP for replicating rotavirus vaccines, it may be less relevant for next-generation non-replicating vaccines^38^. Our understanding of B cell, T cell, and mucosal responses remains limited due to sample constraints and technical barriers, though emerging tools like organoids, CHIMs, and mucosal assays may help address these gaps^26,27,39,40^.

Manuel Franco (Instituto de Genética Humana, Facultad de Medicina, Colombia) presented their insights into early-life T cell development using a neonatal mouse rotavirus challenge model. His presentation examined the development, phenotype, and function of rotavirus-specific intestinal tissue-resident memory T cells (iTRM) in early life. The data showed that neonatal T cell responses differ markedly from adult responses, exhibiting short-lived and phenotypically immature characteristics. Consistent with recent findings, unique populations of CD8⁺ T cells and CD4⁺ regulatory T-cells (Tregs) emerge sequentially during early life, forming functional layers that are distinct from those observed in adults^41^. Early CD8+ cells have different receptors and respond more quickly but exhibit less memory than adult cells. Prior to the development of adult Tregs, early Treg populations enable immune tolerance to self-antigens, and middle phase Tregs enable tolerance to the gut microbiome^42,43^.

Importantly, studies using EED mouse models infected with adherent-invasive *E. coli* have shown that immunization with double mutant heat-labile toxin induces expansion of intestine-resident RORγT⁺FOXP3⁺ Treg cells, alongside reduced vaccine-specific CD4⁺ T cell responses compared with healthy controls. Depletion of Treg cells restored vaccine responsiveness in EED mice; however, this was associated with an increased risk of stunting, highlighting a critical trade-off between immune regulation and growth^44^. These findings demonstrated that local Tregs can suppress vaccine-induced mucosal immunity, limiting both CD4 + T cell accumulation and IgA responses, and may represent a key barrier to effective oral vaccine performance in early childhood.

In a neonatal mouse model of rhesus rotavirus vaccination, designed to assess the role of transforming growth factor-β (TGF-β) and latency-associated peptide (LAP), vaccination induced CD4⁺ T cells expressing TGF-β and LAP. Treatment with anti-LAP antibodies enhanced protection against homologous murine rotavirus challenge compared with controls^45^. Rotavirus-specific T cell responses also differ by age: adult mice exhibit an early, transient T cell response during acute infection, whereas neonatal mice display biphasic CD8⁺ T cell responses^46^. Overall, early-life CD8⁺ T cells and CD4⁺ Tregs appear to function as distinct “layers,” with different phenotypes and receptor profiles compared to adult T cells. Consistent with this, early-life rotavirus-specific T cell responses in both the spleen and intestine are short-lived, with intestinal tissue-resident memory T cells exhibiting an immature phenotype.

Sarah Caddy (Cornell University, USA) highlighted recent advances in the understanding of non-neutralizing antibody (nNAb) responses to rotavirus. While many viruses induce nNAbs that bind to viral particles without directly preventing replication, these antibodies have been shown to confer protection in animal models, although their mechanisms of action have remained poorly defined. Although VP6 is highly immunogenic, it is located within the viral capsid and is only exposed intracellularly. Notably, anti-VP6 nNAbs have been shown to mediate protection by acting within infected cells, where they interact with double-layered particles (DLPs) and block viral transcription^47^. The presentation challenged the field’s traditional emphasis on nNABs by demonstrating that nNAb-VP6-specific IgA and IgG can mediate intracellular neutralisation of rotavirus. They described their newly developed intracellular neutralization assay in mice that quantifies antibody-mediated inhibition of viral replication within infected cells^27^. This process is mediated by the cytosolic antibody receptor TRIM21, which recognises antibody-virus complexes and triggers intracellular immune defence pathways. Intracellular neutralisation has been shown to confer protection against rotavirus infection in both in vitro and in vivo models, suggesting its potential as a novel CoP in humans. In addition, emerging mouse models of maternal antibody transfer may provide valuable platforms for evaluating next-generation rotavirus vaccine strategies.

Miren Iturriza-Gomara (GlaxoSmithKline Vaccines for Global Health) summarised maternal and host determinants of rotavirus disease susceptibility and ORV performance, focusing on histo-blood group antigens (HBGAs), including Lewis and secretor status, and maternally derived antibodies. Host genetics influence susceptibility: the rotavirus VP4 protein binds HBGAs in a genotype-dependent manner, with Lewis-negative and non-secretor children showing resistance to common P[8] rotavirus strains but susceptibility to P[6] strains^48^. As P[8] is included in Rotarix, this may partly explain reduced vaccine effectiveness in some regions. HBGA distributions vary globally. Secretor prevalence is ~75–80% in Europe, North America, Central Asia, and parts of Africa, but lower in regions such as Saudi Arabia and the Philippines ( > 50% non-secretors). The Lewis-negative phenotype is more common in African populations (20–35%) than elsewhere (6–11%)^49–51^. These variations may shape both infection risk and vaccine response.

Maternal antibodies, transplacental and via breast milk, are key contributors to reduced ORV immunogenicity in LMICs. Observational studies consistently show an inverse relationship between maternal antibody titres and infant ORV responses^18,23,52,53^, although breastfeeding interruption has not improved outcomes^54^. Secretor mothers tend to have higher rotavirus-specific antibody titres, potentially increasing interference; in Bangladesh, infants of secretor mothers had lower IgA seroconversion rates^21^. Human milk oligosaccharides (HMOs), particularly fucosylated forms in secretor mothers, can inhibit P[8] and P[4] infectivity in vitro^55^. However, in the opinion of experts at the convening, associations between HMOs, microbiota (e.g., bifidobacteria), and vaccine response remain inconsistent^56^.

Evidence linking HBGA status to vaccine response is inconclusive. Some studies associate non-secretor status with reduced seroconversion and shedding for Rotarix, while others (RotaTeq, RV3-BB) show no association^20^. Conversely, studies in Malawi and multi-country African settings report reduced clinical vaccine failure among non-secretors^51,57^. Maternal antibody interference appears context-specific: negative correlations with immunogenicity were observed in Malawi and India but not the UK, despite similar maternal antibody levels^18^. Increased microbiota diversity is also linked to reduced immunogenicity in LMICs, and neonatal infection status strongly predicts antibody responses^18^.

Interventions such as withholding breastfeeding or increasing vaccine dose have not improved immunogenicity^58^. Low birth weight and oral polio vaccine (OPV) interference are additional risk factors for vaccine failure^59^.

The panel concluded that potential strategies to improve ORV performance include delayed or booster dosing, neonatal vaccination, separating OPV and ORV schedules, improving nutrition, and exploring microbiome-targeted interventions. Further research on HMOs, maternal factors, and alternative vaccine strategies remains essential. Collectively, these presentations underscored the multifactorial nature of rotavirus mucosal immunity, encompassing nonclassical antibody functions, tightly regulated mucosal T cell responses, host genetic factors and maternal influences. Thus, progress towards more effective vaccines will require moving beyond serum-based correlates, integrating both systemic and mucosal immune insights, and leveraging advanced models and assays to optimize existing vaccines and inform next-generation vaccine design.

### Early life mucosal immune development

Anna George (Translational Health Science and Technology Institute, India) outlined current understanding of mucosal cell-mediated immunity in the gut. In early life, intestinal immunity is developmentally regulated: cathelicidin is expressed neonatally but declines with Paneth cell maturation; immature Paneth cells in preterm infants contribute to susceptibility to necrotising enterocolitis^60^.

Rotavirus binds enterocyte sialic acid and HBGAs, while goblet cell mucins act as decoy receptors. Early-life predominance of sialylated over fucosylated glycans may promote dysbiosis and viral susceptibility^61^. Goblet cell-associated antigen passages (GAPs) deliver antigens to dendritic cells (DCs), promoting regulatory T-cell (Treg) development in early life^62^. Infant-derived intestinal organoids show reduced barrier integrity, lower IFN-λ responses, and higher permeability compared to adults^63^. Microbial metabolites such as short-chain fatty acids shape immunity; maternal diet influences neonatal DC development via Flt3L signalling, while antibiotics disrupt this pathway^64^. Batf3-dependent DCs are essential for antiviral CD8 and CD4 responses^65^. Neonatal T-cells are less diverse, skewed toward regulatory phenotypes, and exhibit limited memory.

Tissue-resident memory T (Trm) and B (Brm) cells provide rapid mucosal protection, though their development is context-dependent^66^. Maternal infection can enhance offspring immunity but increase inflammatory risk^67^ Vaccine strategies must balance regulatory and effector responses, prioritise mucosal delivery to induce Trm cells, and consider adjuvants and gut-homing mechanisms^68^.

For rotavirus, comparing neutralizing (NAb) and non-neutralizing (nNAb) antibody responses following natural infection and vaccination in children from LMICs and HICs, alongside detailed mapping of VP4 and VP7 epitopes, may help define CoPs. Cross-reactive antibodies are likely important for addressing the greater viral diversity observed in LMICs. Mucosal immune responses can be assessed using synthetic absorptive matrices (SAM) to sample mucosal lining fluid, while T and B cells can be isolated from mucosal samples using specialised “fluffy” swabs^69^. Collectively, these presentations highlighted several recurring principles of mucosal immunity across pathogens:

- The essential role of tissue-resident memory cells (TRMs) in sustaining durable mucosal immune protection.
- The influence of maternal antibodies on shaping early-life vaccine responses.
- The need to prioritise site-specific mucosal IgA as a key CoP.

We conclude that these lessons reinforce the importance of developing rotavirus vaccines that effectively stimulate local mucosal immunity while accounting for maternal and environmental determinants that influence oral vaccine performance.

### Lessons from other pathogens

Several sessions explored immune responses to other enteric, respiratory, and systemic pathogens to identify cross-cutting insights relevant to mucosal immunity and to improving rotavirus vaccine performance.

### Immune responses associated with infections of the gut mucosa

#### Vibrio cholerae

Edward Ryan (Harvard Medical School, USA) reviewed cholera vaccines and mucosal immune responses to *Vibrio cholerae* infection. Cholera remains endemic in over 40 countries, causing tens of thousands of deaths annually, and shares key features with Rotavirus: both are mucosal enteric infections causing severe diarrhoea in low-resource settings, with reduced vaccine efficacy in young children in LMICs (17–35% failure)^70^. Unlike rotavirus, V. cholerae is a non-invasive extracellular bacterium with an environmental reservoir^71^. Only serogroups O1 and O139 cause epidemic disease. Following ingestion, bacteria that survive gastric acid colonise the small intestine, where they upregulate virulence genes, form microcolonies, and secrete cholera toxin (CT). CT induces chloride secretion via cAMP activation, leading to profuse diarrhoea. After 6–12 hours, bacteria detach and are shed in a hyperinfectious state^72^.

Natural infection induces mucosal immunity lasting 6–10 years, even in children with protection mediated by secretary IgA (sIgA) and IgM targeting bacteria in the intestinal lumen. Protection is serogroup-specific and directed against the O-specific polysaccharide (OSP). Current killed oral cholera vaccines (kOCVs), based on OSP/lipopolysaccharide, show limited efficacy in children under five ( ~ 30%, short-lived) compared to 65–80% in older individuals^73^. In contrast, natural infection provides 85–90% protection for 3–10 years. OSP is a T cell independent antigen, limiting responses in young children. Infection induces strong innate and mucosal inflammatory responses (e.g., IL-23, IL-17, IL-8, NF-κB, MAPK activation)^74^, partly driven by CT’s adjuvant properties. Children mount robust OSP responses after infection but weaker responses after vaccination, with reduced T cell memory and downstream B cell responses^75,76^. Mucosal-associated invariant T (MAIT) cells may partially compensate for T cell independent responses^77^. Intestinal IgM provides short-term protection, but OCVs fail to induce sustained anti-OSP responses in young children. Ryan noted that although CT is internalised during infection, anti-CT antibodies do not confer protection. Instead, protective immunity is driven by functional antibodies that inhibit bacterial motility and colonisation^78,79^. He further discussed how innate immune activation in the gut shapes adaptive responses, and how host factors—including blood group, microbiota, enteric parasites, and EED influence vaccine performance. Notably, innate immune activation associated with EED may enhance OCV responses. Key lessons include prioritising protective (not just immunogenic) antigens, recognising the functional role of low-affinity antibodies and sIgA in inhibiting motility, and understanding modifiers such as blood group, microbiota, co-infections, and EED, which may enhance innate responses^78,79^. These insights are highly relevant for improving enteric vaccines, including rotavirus.

#### Poliovirus

Elizabeth Brickley (London School of Hygiene & Tropical Medicine, UK) highlighted key distinctions between systemic and intestinal immunity using poliovirus as a model. Poliovirus, a single-stranded RNA enterovirus with three serotypes, replicates in the oropharynx and intestine, with <1% of infections leading to paralytic disease. The inactivated polio vaccine (IPV) induces strong systemic immunity that prevents paralysis, while the oral polio vaccine (OPV) additionally induces robust mucosal immunity, interrupting transmission through intestinal replication and secondary spread. OPV has been central to the Global Polio Eradication Initiative, reducing incidence by >99.9% since 1988, although vaccine-derived strains can emerge in under-immunised populations^80^.

Vaccine challenge studies demonstrate that mucosal immunity is replication-dependent. Brickley showed that stool shedding is strongly reduced following OPV but not with IPV, indicating that IPV does not effectively induce intestinal immunity^81^. Additional IPV doses increase serum neutralising antibodies but do not reduce viral shedding or enhance mucosal responses^82^. Both intestinal neutralization and IgA-mediated binding require active viral replication, underscoring the importance of replication-dependant priming.

Age is a critical determinant: OPV induces stronger mucosal immunity when administered in infancy. In children and adult previously vaccinated with IPV, mucosal neutralization responses following OPV challenge are limited, with lower responses observed in adults^83^. The novel OPV type 2 (nOPV2) induces mucosal immune responses comparable to Sabin mOPV2 in infants and neonates, though responses in neonates are slower to develop^84^. In contrast, IPV can induce nasopharyngeal mucosal immunity correlated with systemic responses but has minimal impact on intestinal immunity^85^. In addition, Nicolas Grassly (Imperial College London, UK) compared mucosal immune responses elicited by IPV and OPV, noting that while IPV can induce nasopharyngeal mucosal neutralization, it provides little to no enhancement of intestinal immunity, even with increased dose or potency.

Overall, these findings show that effective intestinal immunity depends on live, replicating vaccines, with IgA playing a central role. OPV remains the most effective approach for inducing mucosal protection, particularly when administered early in life, while nOPV2 offers comparable mucosal immunogenicity with potential programmatic advantages.

### Mucosal immune responses to respiratory tract infections

#### SARS-CoV-2, RSV and GAS

Adam Finn (University of Bristol, UK) discussed mucosal immunity to respiratory pathogens such as severe acute respiratory syndrome corona virus 2 (SARS-CoV-2), respiratory syncytial virus (RSV) and Group A Streptococcus (GAS), highlighting fundamental differences between mucosal and systemic immunity^86^. While serum neutralising antibodies correlate with protection against severe disease, they do not fully prevent infection or reinfection; instead, mucosal IgA and tissue-resident immune responses are critical. Repeated mucosal exposure, as seen in GAS, shapes immune memory, inflammation, and susceptibility, especially in children. His presentation emphasised that immune protection at mucosal surfaces is highly compartmentalised, context-specific and often uncoupled from systemic correlates.

Vaccines can also provide indirect effects, such as reducing transmission through decreased mucosal carriage, which may exceed direct protection. Polysaccharide-protein conjugate vaccines have been shown to induce mucosal responses, including salivary antibodies^87^. Following the introduction of the MenACWY vaccine during a Meningococcal Group W outbreak in England, reduced meningococcal carriage demonstrated potential herd immunity effects^88^. Emerging technologies, such as oral mucosal delivery patches for antibody-based biologics, may further enhance mucosal protection^89^. These findings emphasise that effective vaccines - particularly for enteric pathogens like rotavirus should target mucosal immunity to reduce both disease and transmission. Jinal Bhiman (National Institute for Communicable Diseases, South Africa) expanded on mucosal immunity using SARS-CoV-2 and HIV, focusing on measurements and functional interpretation of mucosal antibodies^90,91^. Studies of genital and respiratory mucosa demonstrated that IgG often predominates over IgA, reflecting transudation from serum, yet retains functional neutralization capacity. In HIV, broadly neutralizing antibodies (bNAbs) target conserved Env regions and reduce viral load, with similar IgA:IgG ratios observed in genital mucosa and plasma despite higher systemic titres^92^.

For SARS-CoV-2, mucosal IgA and IgG responses correlated with reduced reinfection risk, although systematic antibodies alone do not fully explain protection and wane over time^93^. Antigen-specific antibodies are detectable at mucosal sites, but interpretation depends on sample quality. Key challenges include sampling constraints, poor standardisation, and limited assay sensitivity, which continue to hinder progress in mucosal immunology.

#### Group B Streptococcus

Gaurav Kwatra (Cincinnati Children’s Hospital Medical Center, USA) summarised the role of maternal antibody transfer in protection against Group B Streptococcus (GBS)^94^. GBS, a common intestinal and vaginal commensal, is a leading cause of neonatal infection, with maternal colonisation increasing risks of preterm birth, stillbirth, and infant disease. Approximately 50% of infants of colonised mothers acquire GBS, though only ~2% develop early-onset disease. Maternal colonisation prevalence and serotype distribution vary globally^95^. Serotype-specific maternal IgG strongly correlates with mucosal IgG and infant protection, supporting cord blood antibody thresholds as CoP^96^. A hexavalent maternal vaccine (GBS6) induces protective antibody transfer to infants^97^. These findings support the licensure pathways based on immunological correlates rather than large-scale efficacy trials for pathogens associated with relatively rare but severe disease outcome. Ongoing work focuses on defining IgG thresholds, understanding mucosal and breast milk immunity, and developing next-generation vaccines^98^.

### What can gut immunology learn from immune responses to systemic infections?

Satyajit Rath (Indian Institute of Science Education and Research) outlined how systemic immune responses inform gut immunology. Effective protection involves NAbs, memory B cells, CD4⁺ helper and inflammatory T cells, and cytotoxic CD8⁺ T cells. Optimising B cell responses is critical to prevent immune escape. Systemic infection can arise from mucosal spillover (e.g., rotavirus), and memory CD8⁺ T cell competence depends on exposure frequency and timing^99^. T cell biology is shaped by tissue residency, metabolism, Tregs, and non-immune signals^100^. In mesenteric lymph nodes, retinoic acid imprints gut-homing properties, enabling formation of Trm cells, unlike spleen-primed cells^101^. Metabolic pathways influence fate: glutamine metabolism promotes terminal effector differentiation, while its inhibition favours memory formation^102^. Treg-derived IL-10 supports memory differentiation, and neural circuits (liver–brain–gut) maintain peripheral Tregs^103^.

#### Bacillus Calmette-Guérin (BCG) vaccine

Systemic vaccines such as BCG can induce broad immunity, including circulating and tissue-resident memory and cross-protection^104^. Cancer vaccine studies similarly show that systemic immunization can generate CD8⁺ T cells that home to distant tissues^105^. These findings suggest that parenteral vaccines, combined with adjuvants promoting gut homing, may enhance protection against enteric infections.

#### Ebola virus and SARS-CoV-2

Georgios Pollakis (University of Liverpool) presented work from his group on molecular tools for studying humoral immunity. Pseudovirus particles (PVPs) mimic viral entry and enable quantification of neutralizing antibodies via reporter signals, revealing phenomena such as antibody recrudescence in Ebola survivors and enabling SARS-CoV-2 antibody measurement from dried samples^106,107^. Advanced quantitative methods, including TaqMan assays for HIV DNA/RNA and the Blink X microsphere platform, allow sensitive, multiplexed detection^108^. Virus-like particles (VLPs), composed of structural proteins without genetic material, represent promising vaccine platforms capable of eliciting strong immune responses^109,110^.

#### Human immunodeficiency virus (HIV)

William Paxton (University of Liverpool) highlighted how mucosal secretions modulate HIV-1 transmission. HIV crosses mucosal barriers via multiple routes, infecting dendritic cells and CD4⁺ T cells. Breast milk inhibits viral transfer through components such as bile salt stimulated lipase (BSSL), while mucin 6 and lactoferrin in bodily secretions bind DC-SIGN, blocking viral capture and transfer^111^. Variability in these molecules influences transmission and disease progression. These findings underscore the importance of mucosal factors in shaping infection and vaccine outcomes, with relevance for rotavirus.

#### CD8⁺ T-cell responses

Kondwani C. Jambo (Malawi Liverpool Wellcome Research Programme) emphasised the importance of tissue-specific CD8⁺ T cell responses. CD8⁺ T cells vary in function, abundance, and recirculation across tissues, with Trm cells showing site-specific adaptations. Cytolytic CD8⁺ T cells are largely confined to circulation^112^. Studies of duodenal tissue in Malawian adults revealed predominantly Trm CD8⁺ T cells with limited cytolytic capacity and marked spatial and functional heterogeneity^113^. CD103 expression defined subsets, while KLRG1⁺ cells exhibited higher cytotoxic potential.

Findings from mucosal and systemic infections and vaccine responses demonstrate how peripheral blood measurements alone are insufficient to capture mucosal immunity. A deeper understanding of local immune responses, particularly Trm dynamics, is essential for advancing vaccines against enteric pathogens such as rotavirus.

### Model systems

This session highlighted innovative experimental systems that could transform the study of rotavirus infection and immunity.

#### Organoid models

Sasirekha Ramani (Baylor College of Medicine, USA) described their work on human intestinal enteroids (HIEs) as advanced models for studying gut infections. Derived from intestinal crypt stem cells, HIEs are non-transformed epithelial organoids capable of indefinite passaging and comprising all key epithelial cell types (enterocytes, goblet, enteroendocrine, and Paneth cells)^114^. They retain physiological structure and function, including lumenal expansion during rotavirus infection, mimicking diarrhoeal processes^40^. HEIs can be used to investigate host susceptibility, viral pathogenesis, and factors contributing to vaccine interference^40^. HIEs enable investigation of host genetic susceptibility. Norovirus infection depends on HBGAs regulated by FUT2; secretor derived HIEs are susceptible, while non-secretors are resistant. Similar patterns are observed for rotavirus, where FUT2 manipulation alters susceptibility^40^. HIEs also support neutralization studies, including analyses of VP8* antibody contributions and variability across populations^115^, also noted the important differences between infant- and adult-derived HIEs in innate immune responses and epithelial barrier function, underscoring the importance of age-appropriate models for understanding infant rotavirus disease.

These models replicate vaccine-relevant phenomena, including attenuated phenotypes and interference effects. Co-infection studies show reduced rotavirus vaccine shedding when OPV or other enteroviruses are present. Infant-derived HIEs differ markedly from adult models, with altered transcriptional profiles, increased permeability (e.g., Claudin-2 expression), and reduced interferon responses^63^. HIEs from children with EED show reduced rotavirus replication. Current advances include integrating immune cells and microbiota through oxygen-gradient co-culture systems.

#### Controlled human infection models (CHIMs)

Ben Morton (Liverpool School of Tropical Medicine, UK) and Michelo Simuyandi (Centre for Infectious Diseases Research, Zambia) demonstrated how integrating CHIMs with advanced immune profiling can greatly enhance our understanding of rotavirus mucosal immunity and vaccine performance. Drawing on pneumococcal CHIM experience in the UK^116^, Morton described how systemic serology can be used to interrogate antibody quality and functional activity, rather than titres alone, to identify immune features associated with protection and discussed how CHIMs can be utilised as tools for vaccine evaluation and immunological discovery. Their experimental human pneumococcal challenge (EHPC) model enables detailed study of colonisation, immune responses, and CoP through controlled exposure and intensive sampling^116–118^. EHPC studies demonstrated that PCV13 provides 78% protection against pneumococcal colonisation and reduces carriage density and duration^119^. The model has also been applied in Malawi, where vaccine effectiveness against carriage was lower (62%), helping explain reduced herd immunity in low-income settings^120^. Ongoing work addresses vaccine escape serotypes, including serotype 3, and is evaluating PCV13 and PPV23 efficacy^121^. CHIMs also enable investigation of co-infection dynamics. Influenza-pneumococcal co-infection increases inflammation and impairs monocyte recruitment, while incidental viral infections, particularly RSV, increase pneumococcal carriage^122,123^. Current trials are exploring pathogen interactions and immune responses in RSV co-infection.

Simuyandi presented preliminary findings from a rotavirus CHIM in 720 Zambian infants, evaluating oral (Rotarix), parenteral VP8* subunit, and combined regimens. Approximately 180 infants per arm were stratified by HIV exposure and OPV status. The study assessed vaccine-induced protection via viral shedding (qPCR days 5, 7, 9) and immune responses^124^. He showed that infant CHIMs in these settings are both feasible and informative, and that intestinal endpoints such as viral shedding reveal differences between vaccination strategies that are not apparent from serum antibody measurements alone. Combined vaccination was safe and well tolerated, with no major differences in adverse events between schedules. The parenteral-only arm showed higher shedding and viral loads compared to oral and combined arms. IgA seroconversion was ~50% in oral and combined groups but lower ( ~ 20%) in the parenteral arm. However, parenteral vaccination induced stronger B cell activation (CD38) and gut-homing responses (α4β7). Combined prime-boost regimens enhanced these responses. Ongoing analyses include neutralization, VP6-specific immunity, and systems immunology approaches to identify CoPs. Together, organoid and CHIM models provide complementary platforms to dissect host-pathogen interactions, evaluate vaccines, and define CoP, particularly for enteric infections such as rotavirus.

#### Emerging experimental models

Steeve Boulant (University of Florida, USA) and Siyuan Ding (Washington University in St. Louis) further highlighted next-generation experimental platforms, including organoid-microbiome co-culture models that simulate physiological conditions such as hypoxia and dysbiosis^125^ and reverse genetics^126^. They demonstrated how the gut’s physiologic hypoxic environment shapes host-pathogen interactions. The intestinal epithelium exists along a steep oxygen gradient, from the anoxic lumen to the oxygen-rich subepithelium, enabling tolerance to commensals while maintaining pathogen responsiveness. Disruption of this balance can lead to inflammation and disease. These systems reveal mucosal immune mechanisms that remain undetectable in conventional cell line or animal models and offer valuable insights for rational rotavirus vaccine design. Using rotavirus infection models, Boulant’s group showed that hypoxia suppresses type I and III interferon (IFN) responses by impairing TBK1 and IRF3, key regulators of IFN production, thereby promoting viral replication^127^. This effect is not pathogen-specific but reflects a broader property of the gut immune environment. In mouse models, rotavirus infection is restricted to villus tips, where hypoxia limits IFN responses, while normoxic crypts mount stronger antiviral responses^128^. Type III IFN is critical for protection, and hypoxia-driven suppression appears linked to PP2A-mediated regulation of TBK1/IRF3 signalling^129^. Similar hypoxia-associated immune suppression is observed across viral infections. These findings highlight hypoxia as a key regulator of mucosal immunity and suggest that modulating oxygen dynamics could enhance vaccine replication and effectiveness.

#### Reverse genetics

Siyuan Ding presented advances in rotavirus-vectored dual vaccines using reverse genetics. Given the limited ability of parenteral vaccines in eliciting mucosal IgA, rotavirus vectors offer a promising platform for oral vaccines to elicit mucosal IgA, rotavirus vectors offer a promising platform for multivalent oral vaccines targeting multiple enteric pathogens. A plasmid-based reverse genetics system enables recovery of recombinant rotaviruses using helper plasmids that enhance replication and translation efficiency^126^. A third-generation system allows generation of highly attenuated strains, while CRISPR/Cas9 screening has identified host factors (SERPINB1, TMEM236) that influence viral replication. Using this platform, recombinant rotavirus expressing human norovirus VP1 induces strong systemic and mucosal antibody responses, supporting its use for antigen delivery^130^. Dual rotavirus-based vaccines incorporating enterotoxigenic and uropathogenic *E. coli* antigens (e.g., heat-labile toxin and FimH) demonstrate promising immunogenicity, with enhanced responses following inclusion of LT-B. Importantly, insertion of heterologous antigens does not compromise rotavirus-specific IgA responses, highlighting the potential of this platform for next-generation combination enteric vaccines.

Collectively, these insights reinforce the importance of live replication in generating durable mucosal immunity, a principle directly relevant to understanding and improving ORV performance. Parenteral vaccines can protect against severe disease but offer limited protection against infection, as they induce minimal secretory IgA at mucosal surfaces. This highlights the need for effective mucosal vaccination platforms, potentially building on existing vaccines. Rotavirus reverse genetics provides a powerful tool for antigen delivery, although its genome has limited coding capacity. Smaller antigens can be incorporated into different vectors, as co-delivery does not require both antigens to enter the same cell. This strategy shows promise but may require careful optimisation of dosing regimens. Together, these emerging models provide powerful, complementary tools for dissecting mucosal immune mechanisms and guiding the development of next-generation oral and vectored rotavirus vaccines.

### Maternal-infant immunity and correlates of protection—critical understanding and role in vaccine development

This session emphasised the dual role of maternal antibodies as both protective and inhibitory for ORV responses^39,52^. Sasirekha Ramani and Georgia Tomaras (Duke University, USA) highlighted how maternal antibodies can dampen ORV immunogenicity and complicate the identification of CoPs, drawing parallels with challenges encountered in HIV-1 vaccine efficacy trials. They underscored the importance of longitudinal maternal-infant cohort studies to characterize antibody transfer and define mechanisms of early life protection.

Anna George and Jinal Bhiman further explored mucosal and cell-mediated gut immunity, noting that maternal antibodies may interfere with these responses. They emphasized the need for mechanistic models to disentangle the roles of neutralising, Fc-mediated, and intracellular antibody functions in vaccine effectiveness^131^. Insights from studies of GBS presented by Gaurav Kwatra demonstrated the feasibility of defining serological CoPs. Evidence from natural infection and previous vaccine studies suggests that maternal serum-specific IgG correlates protective against colonization and disease and higher mucosal IgG may reduce colonization risk^94,131^. These findings support maternal antibody thresholds as CoPs^97,98,131^, although maternal antibody interference remains a key limitation for ORV performance^39^.

Vineeta Bal reviewed CoPs in systemic infections, noting their complexity due to pathogen diversity and routes of exposure. CoPs may be measured after infection, vaccination, or both, and include direct immunological markers (e.g., neutralizing antibodies, homing receptors, tissue-resident cells) and indirect omics-based signatures. Systems approaches, such as cytokine networks, can capture organism-wide immune responses^132^. Differences in vaccine performance between LMICs and HICs are more evident for enteric than respiratory infections. During COVID-19, neutralizing antibodies initially served as CoPs, but their predictive value declined with viral evolution and waning immunity^133^. Broader strategies, such as mosaic nanoparticle vaccines targeting conserved epitopes, show promise for cross-protection^134^. These advances have informed tuberculosis vaccine development, where CoPs may differ for infection, disease, and transmission, with mucosal immunity likely central^135^. Pre-vaccination immune “endotypes” may also predict vaccine responsiveness^136^.

Georgia Tomaras highlighted lessons from HIV vaccine trials, where protection depends on coordinated humoral and cellular responses^137^. Correlates include antibody specificity, isotype, subclass (e.g., V2 IgG3), and Fc function, with complex, multifactorial CoPs emerging from integrated analyses^138,139^. Antibody immunoprophylaxis studies have further identified neutralization and concentration thresholds as CoPs^140^.

Overall, CoP are likely multifaceted, integrating systemic and mucosal immunity, host factors, and pathogen diversity. Advancing rotavirus vaccine development will require moving beyond serum IgA, incorporating mucosal sampling, high-dimensional omics, and computational modelling to define protective immunity and guide next-generation vaccine design^141^.

## Discussion

The convening in Liverpool underscored the urgent need for an integrated research agenda that links mechanistic immunology with translational vaccine development. Insights from other pathogens reinforced the importance of maternal antibody studies, characterizing tissue-resident memory cells, and conducting site-specific mucosal sampling to define protective immunity. Emerging experimental platforms, including HIEs, CHIMs and reverse genetics, offer unprecedented opportunities to investigate the biological drivers of ORV underperformance. Yet significant challenges remain, including limited access to these platforms, variability in assay performance, and lack of standardization, particularly in LMICs. Addressing these gaps will require strengthened collaborative networks, harmonised methodologies, and robust data-sharing frameworks. Improving ORV performance, especially in LMICs, demands a deeper understanding of the immune mechanisms operating at the intestinal mucosa, where protection is ultimately mediated. Although ORVs have contributed substantially to reductions in global childhood mortality, their effectiveness remains lower in LMIC settings due to a complex interplay of host, pathogen, and environmental factors^142–145^.

A central theme was the pivotal role of mucosal immunology in shaping rotavirus vaccine performance. Participants emphasized that while serum IgA remains the best available surrogate CoP, it is neither sufficient nor mechanistic. Several immunological studies demonstrate that rotavirus protection involves a combination of humoral, cellular, and innate mechanisms that are not adequately captured by serum IgA alone^17,32^. Presentations highlighted emerging roles for Fc-mediated antibody functions, intracellular antibody-mediated immunity, and TRM T cells—mechanisms known to contribute to immunity against other viral pathogens^146,147^. Comparative insights from poliovirus and cholera research underscored the importance of mucosal IgA and live replication vaccines in inducing protective intestinal immunity^78,79,83–85,148^. Similarly, evidence from GBS demonstrates the feasibility of defining maternal antibody thresholds as CoP^131^.

Applying similar approaches to rotavirus could accelerate the identification of robust immune markers but will require harmonised mucosal assays, mechanistic studies, and longitudinal maternal-infant cohorts to define meaningful correlates that can guide next-generation vaccine designs. The roadmap (depicted in Supplementary Figure), articulated at the meeting, prioritizes mechanistic research, longitudinal maternal-infant studies, and novel vaccine strategies as essential steps toward reducing the global burden of rotavirus disease.

A major theme of the convening was the transformative potential of new experimental platforms. HIEs and organoid-microbiome co-culture systems now allow for detailed, physiologically relevant investigation of rotavirus infection and host immune responses^40,115^. These systems are particularly valuable because they can model environmental conditions that contribute to ORV underperformance in LMICs, including malnutrition, dysbiosis, and epithelial hypoxia. CHIMs, including the newly established rotavirus CHIM in Zambia, offer complementary opportunities to assess vaccine performance, mucosal immunity, and viral shedding under real-world conditions^116,124^ and have explored ethical and participant considerations in qualitative studies^149^. Notably, early CHIM findings indicate that intestinal endpoints such as viral shedding provide more informative measures of mucosal immunity than serum antibody titres alone, consistent with the need for mucosal CoP. Despite these advances, access to HIE and CHIM platforms remains limited. Many LMIC researchers, the very groups whose populations are most affected by rotavirus, do not yet have equitable access to these technologies. Expanding availability of HIE lines, building CHIM capacity in additional LMIC settings, and establishing shared protocols will be essential for generating contextually relevant data.

Maternal-infant immunity emerged as both a major challenge and a key opportunity. Maternal antibodies can inhibit ORV replication and reduce vaccine immunogenicity, complicating early-life vaccination strategies^39^. At the same time, maternal immunization has been highly effective for other pathogens such as GBS, influenza, and pertussis^131,141^. Longitudinal maternal-infant cohorts are needed to clarify mechanisms of protection versus interference, delineate maternal antibody decay kinetics, and define quantitative antibody thresholds that correlate with infant protection. Systems immunology and multi-omics approaches—including transcriptomics, epigenetics, and metabolomics—offer promising tools to uncover immune signatures that may serve as CoP^141^. This work will require strong collaboration between institutions in LMICs and HICs to ensure robust and equitable scientific progress.

Across the meeting, several cross-cutting priorities emerged that participants deemed essential for advancing rotavirus immunology and vaccine development. A consistent message was the need to standardize mucosal immunoassays, including harmonised sampling approaches, shared analytical protocols, and comparable reporting frameworks, to enable meaningful integration of data across studies and settings. Participants also highlighted the importance of identifying mucosal CoP beyond serum IgA, incorporating functional antibody responses, TRM T cells, and epithelial-innate immune interactions. Integrating systems immunology approaches, such as transcriptomic, metabolomic, and high-dimensional serological analyses, was viewed as critical for defining mechanistic signatures of protection and for improving the ability to predict vaccine outcomes. Finally, the group stressed the potential of innovative vaccination strategies, including heterologous schedules and parenteral–oral prime–boost regimens, to overcome the inherent limitations of oral vaccines in LMICs. Together, these priorities outline a coordinated path toward a deeper mechanistic understanding of rotavirus immunity and the development of more effective next-generation rotavirus vaccines. Table 1 presents the cross-cutting priorities that emerged that are essential for advancing rotavirus immunology and vaccine development.

**Table 1:** Integrated research priorities for advancing rotavirus immunology and vaccine development

Research domain	Key findings from the convening	Research gaps	Priority actions / next steps	Implications for vaccine development
Mucosal immunity & correlates of protection	Serum IgA correlate weak; Mucosal mechanisms poorly defined TRM cells, Fc-mediated antibodies, epithelial responses likely critical	Lack of standardised mucosal assays; Unclear role of TRM and Fc-mediated antibodies; Limited understanding of functional immune mechanisms	Develop harmonised mucosal sampling and assays; Define correlates beyond serum IgA; Incorporate functional immune readouts	Identification of reliable correlates of protection that reflect intestinal immunity and improve vaccine evaluation
Cross-pathogen insights	Maternal IgG transfer (GBS), mucosal IgA (SARS-CoV-2, RSV), tissue-resident memory (cholera, RSV) are critical correlates	Limited translation of site-specific mucosal correlates into rotavirus research	Apply cross-pathogen methodologies and sampling frameworks to rotavirus studies	Accelerates identification of relevant immune mechanisms and avoids duplication of discovery efforts
Models & systems biology	HIEs, and CHIMs enable mechanistic insights into infection and immunity	Limited access in LMICs; Lack of standardised outputs; Insufficient integration of multi-omics data	Expand CHIMs (especially in LMICs); share HIE platforms; integrate transcriptomic, metabolomic, and systems serology approaches	Enables mechanistic understanding of vaccine responses and supports prediction of efficacy across settings
Maternal–infant immunity	Maternal antibodies influence infection risk and interfere with ORV responses	Mechanisms of interference unclear; optimal timing and dosing strategies undefined	Conduct longitudinal birth cohort studies; Map maternal antibody transfer and infant immune development	Supports optimisation of vaccination schedules and strategies for early-life protection
Vaccine strategies	Oral–parenteral prime–boost and heterologous approaches show promise for improving responses	Limited evidence on durability, optimal combinations, and efficacy endpoints	Evaluate heterologous and adjuvanted vaccine strategies in clinical and experimental studies	Informs development of next-generation vaccines with improved performance in LMICs
Systems integration (cross-cutting)	Multi-omics and high-dimensional analyses can define immune signatures of protection	Fragmented data and lack of integration across studies	Standardise data collection and reporting; Apply systems immunology frameworks across studies	Improves prediction of vaccine outcomes and supports rational vaccine design

*IgA* Immunoglobulin A, *RSV* Respiratory Syncytial Virus, *SARS-CoV-2* Severe Acute Respiratory Syndrome Coronavirus 2, *GBS* Group B Streptococcus, *HIEs* Human Intestinal Enteroids, *CHIMs* Controlled Human Infection Models, *LMICs* Low-and-Middle Income Countries, *TRM* Tissue-Resident Memory T cells, *ORV* Oral Rotavirus Vaccine

A major strength of the convening was its multidisciplinary composition, bringing together experts in virology, mucosal immunology, vaccinology, and clinical research, with strong representation and leadership from LMICs. The meeting effectively integrated cross-pathogen insights and highlighted emerging models such as CHIMs and HIE platforms. Several limitations should be noted. First, conclusions were based on expert presentations and consensus discussions rather than a systematic synthesis of evidence; a formal meta-analysis of mucosal immunity across rotavirus and other pathogens could potentially define knowledge gaps and priorities in detail. Second, many promising assays and models discussed remain early-stage and are not yet validated for large-scale or deployment in LMIC settings. Third, much of the mechanistic evidence is derived from high-income settings, with limited data from LMICs where vaccine underperformance is greatest. Finally, implementation of the proposed roadmap will require sustained resources, coordination, and equitable access to research infrastructure. Addressing these gaps will require global investment, inclusive partnerships, and targeted efforts to strengthen mechanistic research in LMICs.

## Concluding remarks

This expert convening underscored the critical need to advance mucosal immunology research to improve rotavirus vaccine performance. Although global mortality due to diarrhoea in the under-five-year-old children has declined^150^, ORVs continue to underperform in LMICs, revealing significant gaps in understanding the immune mechanisms required for effective protection. Through comparative insights from other mucosal pathogens, advances in experimental models, and new maternal-infant cohort strategies, the convening identified clear priorities for future research. These include harmonising mucosal assays, identifying CoP beyond serum IgA, expanding access to reverse genetics, CHIMs and HIE platforms, and evaluating novel vaccination strategies such as parenteral–oral prime–boost regimens. Delivering on this agenda will require global collaboration, equitable access to scientific tools, and sustained investment from funders and governments. By addressing these scientific and structural gaps, the field can accelerate development of next-generation rotavirus vaccines and move closer to achieving equitable global control of rotavirus disease.

## Recommendations (research roadmap—priorities and gaps)

### Key outcomes and priority areas

ORVs continue to show reduced effectiveness in LMICs, where rotavirus remains a major cause of diarrhoeal mortality in children under five years old. To address this persistent gap, the convening identified five strategic research priorities that funders and international donors may consider for future investment.

#### Optimising vaccine delivery strategies

Alternative delivery approaches should be evaluated to maximize the effectiveness of current vaccines in LMICs. This includes assessing modified dosing schedules, the potential of neonatal administration, and the use of booster doses to strengthen long-term protection.

#### Integrating complementary interventions

Improving gut health and nutritional status was highlighted as a key strategy to enhance vaccine performance. Studies should investigate supplements such as zinc and multiple micronutrient supplements (MMS + ), as well as novel biological interventions including Glucagon-like peptide-2 (GLP2) agonists, to support robust immune responses.

#### Developing next-generation parenteral vaccines

There is strong interest in pursuing parenteral alternatives, such as inactivated whole-virus vaccines, designed with innovative antigen approaches. The inclusion of non-structural proteins and novel adjuvants that stimulate mucosal immunity could expand protective efficacy beyond what current oral formulations achieve.

#### Advancing next-generation oral vaccines

Efforts should focus on improving oral vaccine design to increase stability and immunogenicity. Strategies include optimising formulations to withstand gastric degradation and leveraging reverse genetics or novel cell lines to produce high-titre vaccines capable of eliciting stronger mucosal protection.

#### Standardising tools and models for research and development

The field urgently needs harmonized experimental standards and robust models to accelerate vaccine innovation. Identifying true CoP and advancing platforms such as HIEs, gut-on-a-chip systems, and CHIMs will be critical to improving translational research and guiding next-generation vaccine development.

Collectively, these priority areas form a roadmap to strengthen vaccine performance in LMICs and reduce the global burden of rotavirus disease. Table 1 maps out the key findings from the convening, the research gaps that were identified and what the convening believes are the priority actions and potential next steps to be taken by the research community. Supplementary Fig. 1 maps out how these can priorities can make an impact on reducing the global burden of rotavirus disease.

## Supplementary information

Supplementary

## Data Availability

No datasets were generated or analysed during the current study.
