# Supplementary material for: Rotavirus mucosal immunology: insights and research priorities from an international convening in Liverpool, March 2024
Source: NPJ Vaccines. 2026 Jun 19;11:122. doi: 10.1038/s41541-026-01505-w (PMC13282379; doi:10.1038/s41541-026-01505-w)
Supplement: Supplementary file 1 — Supplementary [file 41541_2026_1505_MOESM1_ESM.pdf]

Supplementary

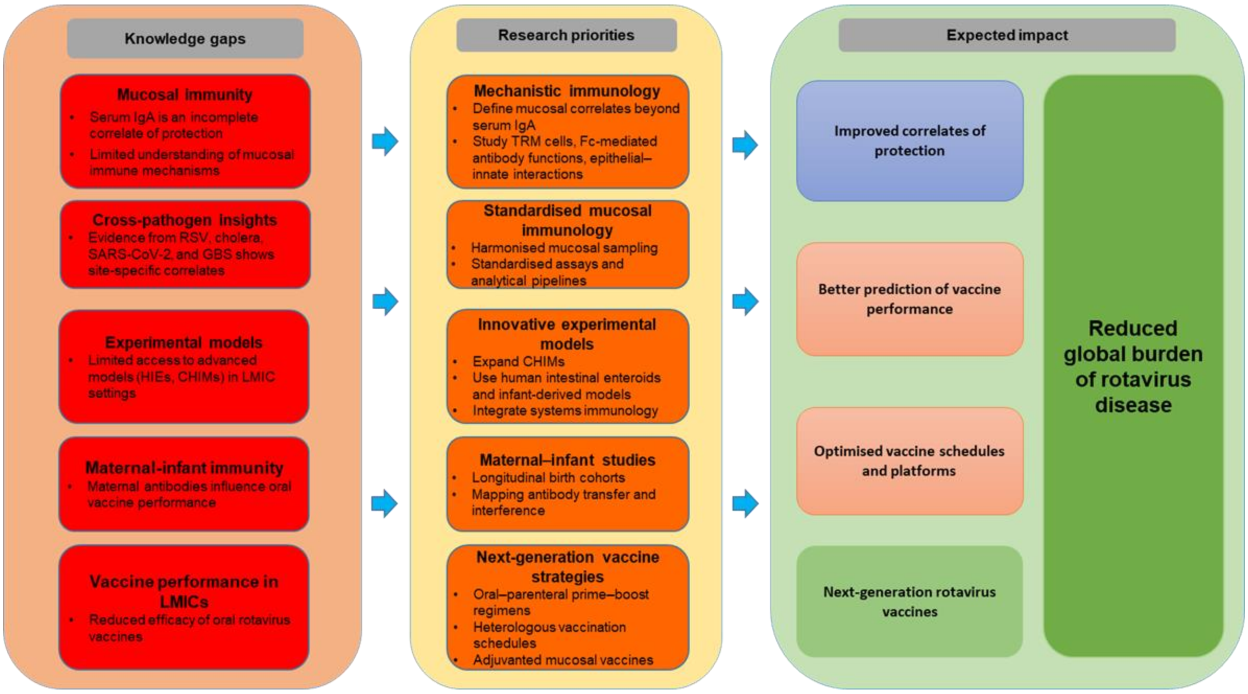

IgA: Immunoglobulin A; RSV: Respiratory Syncytial Virus; SARS-CoV-2: Severe Acute Respiratory Syndrome Coronavirus 2; GBS: Group B Streptococcus; HIEs: Human Intestinal Enteroids; CHIMs: Controlled Human Infection Models; LMICs: Low-and-Middle Income Countries; TRM: Tissue-Resident Memory T cells

Supplementary Figure. Research ecosystem for advancing rotavirus immunology. Key research domains identified during the convening including mucosal immunology, maternal-infant immunity, cross-pathogen insights, experimental models, and innovative vaccine strategies collectively contribute to an integrated mechanistic understanding of rotavirus immunity. This iterative research framework supports identification of correlates of protection, optimisation of vaccine strategies, and the development of next-generation rotavirus vaccines aimed at reducing the global burden of disease.
